# Electromyography activity of the teres minor muscle with varying positions of horizontal abduction in the quadruped position

**DOI:** 10.1016/j.jseint.2020.12.014

**Published:** 2021-02-22

**Authors:** Masaaki Tsuruike, Todd S. Ellenbecker, Connor Lauffenburger

**Affiliations:** a Department of Kinesiology, College of Health and Human Sciences, San José State University, San José, CA, USA; b Rehab Plus Physical Therapy Scottsdale and ATP Tour, Scottsdale, AZ, USA; c Sports Medicine Department, University of Northern Colorado, Greeley, CO, USA

**Keywords:** Horizontal abduction exercise, Quadruped position, Teres minor

## Abstract

**Background:**

The teres minor (TMi) muscle exposed relatively high activity during the acceleration and deceleration phases of the throwing motion, compared with the infraspinatus muscle. However, few studies have identified TMi muscle activity in intervention exercises. The purpose of this study was to investigate TMi muscle activities in different horizontal adduction positions in the quadruped horizontal abduction exercise. This study hypothesized that TMi muscle activity would differ in response to resistance application across different horizontal adduction positions.

**Materials and methods:**

Nineteen collegiate baseball players volunteered their participation. Raw electromyography activity of the TMi muscle along with 7 different muscles attached to the scapula on the dominant-side were collected, and normalized by each of the corresponding maximum voluntary isometric contractions. All subjects performed manual isometric resistance horizontal abduction exercises at 90° and 135° of abduction with 3 horizontal adduction angles in the quadruped position: 1) coronal, 2) scapular, and 3) sagittal plane. Electromyography data were also collected from rhythmical concentric contraction of horizontal abduction at 90° of abduction in the quadruped position.

**Results:**

TMi muscle activity was significantly greater with the arm positioned in the coronal plane than that of the scapular and sagittal planes (41, 26, and 17% maximum voluntary isometric contraction, respectively) (*P* < .05).

**Conclusion:**

The present study demonstrated that TMi muscle activity varied depending on horizontal adduction positions.

The teres minor (TMi) muscle is the smallest of the rotator cuff muscles and characterized as a thick fusiform without a pennate, while the infraspinatus (IS) muscle is composed of a pennate.^2^ External rotation (ER) force synergistically generated by the TMi and IS muscles is cocontracted with the posterior deltoid (PD) muscle.^3^ The activation of the PD muscle increases in ER force as the abduction (ABD) of the glenohumeral joint (GHJ) increases.^24^ In contrast, the tensile force of IS muscle, which was measured in a cadaveric study, decreased as GHJ ABD increased owing to a decrease in the rotational moment arm of the IS muscle, whereas the TMi was not affected by GHJ ABD.^20^ Although the TMi muscle reportedly provides ER force up to 45%,^17^^,^^31^ the TMi muscle may play an important role in ER force in the 90° ABD position.

The amount of IS muscle activity has been demonstrated to be significantly decreased with elbow extension during standing resistive band exercises with the arm positioned at 120° of ABD, compared with the elbow flexed at 90° with the arm positioned at 90° of ABD and ER of the GHJ.^26^ This finding was in line with IS muscle activity that was relatively less than TMi muscle activity during the acceleration and deceleration phases of baseball pitching^12^^,^^14^ in which the elbow joint was extended to 25° at ball release from 110° of flexion which occurred during the late cocking phase of pitching motion along with the maximum external rotation of the GHJ.^13^ Furthermore, a previous study revealed that the mean value of TMi muscle was most highly activated in prone shoulder horizontal abduction (HABD) exercise and as much as side-lying ER exercise among different exercises associated with a baseball rehabilitation program.^25^ However, few studies have investigated TMi muscle electromyography (EMG) activity in intervention exercises compared with the IS muscle across the different horizontal adduction (HADD) positions. Therefore, the purpose of this study was to investigate TMi muscle activity in different arm positions of HADD to determine which arm position produced the highest levels of TMi muscle activity using manual resistance with subjects in a quadruped position. This study hypothesized that TMi muscle activity would vary with the amount of HADD position during quadruped exercise. Because this study used a surface EMG electrode to measure TMi muscle activity, adjacent muscle activities were measured to distinguish any potential contamination of crosstalk signals.

## Methods

During the baseball off-season, 19 male collegiate baseball players belonging to the NCAA D-I conference (height: 181.4 ± 8.1 cm, weight: 86.7 ± 10.0 kg, age: 18.9 ± 1.1 years) volunteered to be examined. All participants gave informed consent to the procedures as approved by the institutional review board of the university before testing. All subjects were asymptomatic, competitive baseball players without neurologic or physiological injuries in the upper body based on the completion of a preliminary screening questionnaire. All tests were performed in the Kinesiology Laboratory.

### Electrode Placement

Raw EMG amplitudes of the TMi, IS, teres major (TMa), PD, middle deltoid (MD), upper trapezius (UT), lower trapezius (LT), and serratus anterior (SA) muscles on the throwing shoulder side were collected. Bipolar surface silver (Ag) EMG electrodes with a bar length of 10 mm, width of 1 mm, and a distance of 10 mm between active recording sites (Delsys Bagnoli-8, Delsys Inc., Natick, MA, USA) were used. The EMG electrodes were preamplified (X 10) and routed through the EMG mainframe, which further amplified (X 100) and band-pass filtered (20-450 Hz) the signals. Electrodes were placed on the center of the muscle belly in line with the muscle fibers for the specific manual muscle test.

**Figure 1:**
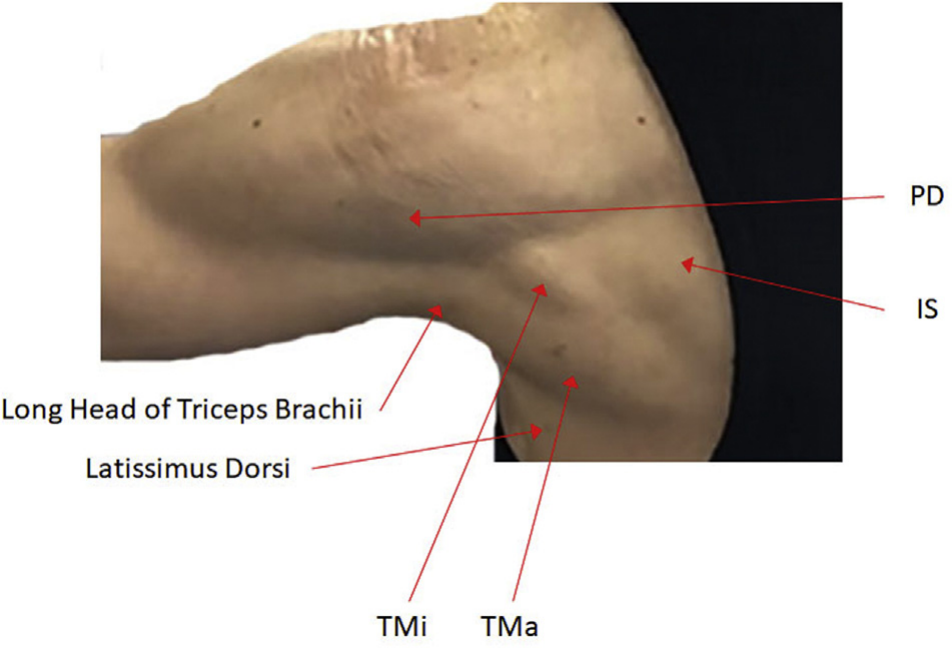
Definition of the teres minor (TMi), infraspinatus (IS), posterior deltoid (PD), teres major (TMa), and latissimus dorsi during horizontal abduction exercise with the elbow extended and shoulder externally rotated while pulling a cable machine.The photo depicts a female subject who did not participate in this study. Typically, the muscular definition of the TMi muscle is unclear in many male subjects who are trained owing to the hypertrophy of the deltoid muscles.

The electrode for the TMi muscle was placed on one-third of the distance from the posterior portion of the acromion process to the inferior angle of the scapula and the lateral aspect of the lateral border of scapula, which was just below the definition of the PD muscle^21^ (Fig. 1). Because surface EMG recordings were used in this study, we presumed that for the theoretical basis of the study, this electrode location was representative of TMi function in our subjects. For the IS muscle, the electrode was placed on inferior and parallel to the scapular spine over the infrascapular fossa,^3^^,^^29^ while the electrode was placed on the lateral aspect of the inferior angle of scapula for the TMa muscle. For the PD muscle, the electrode was placed at an oblique direction parallel to the muscle fibers of the deltoid muscles at the lateral border of the scapular spine;^30^ while the electrode was place at halfway between the tip of acromion and the deltoid tubercle for the MD muscle.^3^^,^^29^ For the UT muscle, the electrode was placed at halfway between the C7 spinous process and the acromion process, while the electrode was placed at an oblique angle from the scapular spine and just outside of the scapular medial border for the LT muscle.^16^ For the SA muscle, the electrode was placed below the axilla between the latissimus dorsi and pectoralis major at the level of the scapular inferior angle.^26^^,^^27^^,^^29^^,^^30^ The reference electrode was placed over the spine of the scapula or between the electrodes of the UT and IS muscle.

### Procedures

Once the electrodes were secured, participants performed a 4-second maximum voluntary isometric contraction (MVIC) after ramp-up contraction for each muscle using the manual muscle strength test (MMT) procedure for normalization of EMG data. The manual pressure was applied by the same examiner for all testing positions to determine each of the MVICs. For the MVICs of the UT and SA, subjects abducted their arms to 90° in the scapular plane with the elbows extended and the thumb pointed toward the ceiling and resisted downward pressure applied on the arm,^28^ whereas for the MD, the MVIC was examined in 90° of ABD of the GHJ with the elbow flexed.^27^ The MVIC of the IS was examined while the subjects resisted the manual pressure applied toward internal rotation of the shoulder with the elbow flexed to 90°, shoulder abducted to 0° and externally rotated to 0°.^22^ The MVICs of the LT and PD were examined in the quadruped position while the subjects elevated their arm to 135° ABD and resisted downward pressure applied on the arm.^27^ The MVICs of the TMi and TMa were examined in the quadruped position at 90° ABD and at 0° of horizontal ABD with the elbow flexed to 90° while the subjects resisted downward pressure applied on the arm.^27^

The amount of force (N) of MMT was determined in both 90 and 135° ABD without ER in quadruped horizontal abduction performed in the coronal plane by the same examiner with a handheld dynamometer (MicroFET, Hoggan Scientific, LLC, Salt Lake City, UT, USA) for each subject. The MMT force was subsequently used to determine the percentage of external load in horizontal adduction.

**Figure 2:**
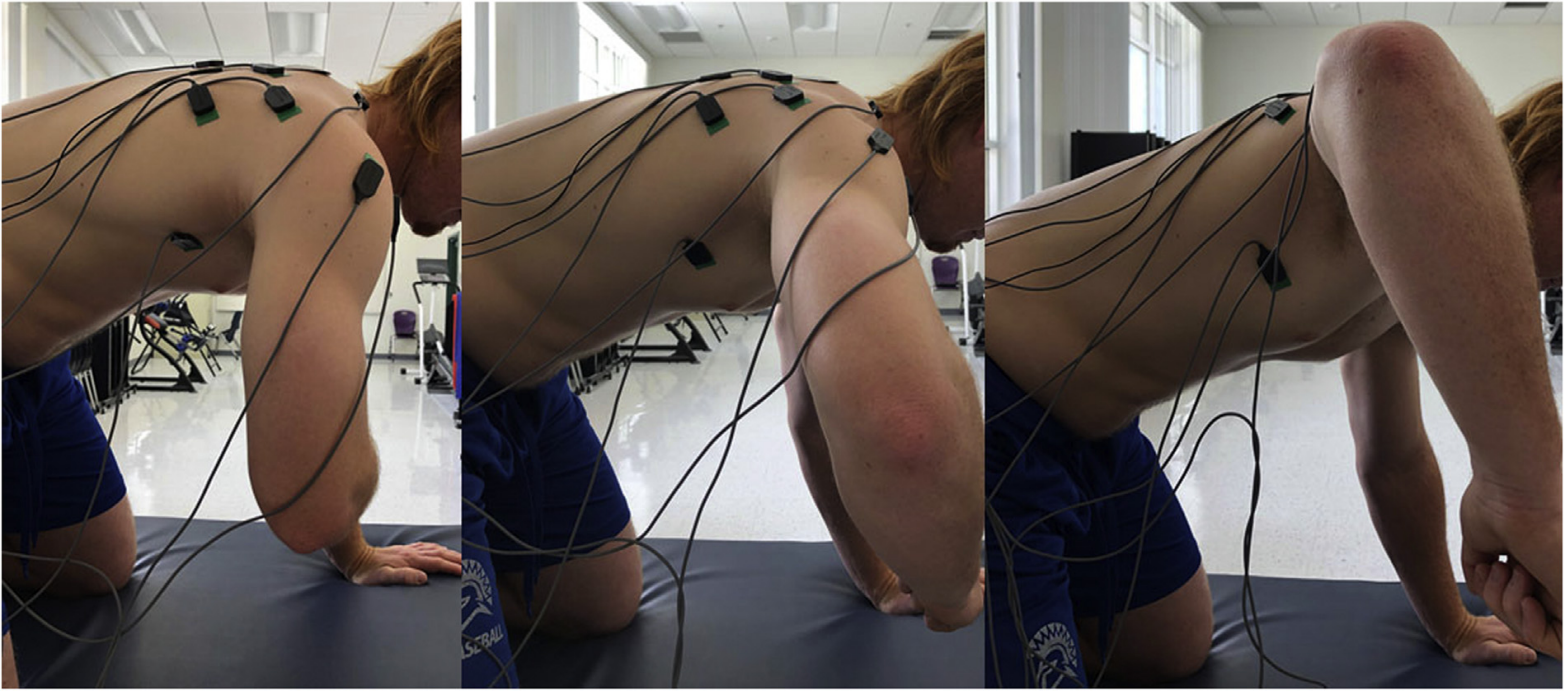
Manual isometric resistance exercise was implemented in the quadruped position. The 3 different arm position angles were shown during the exercise at 0 of abduction of the glenohumeral joint: (left) 90 of horizontal adduction (HADD) or the arm positioned in the sagittal plane, (middle) 50 of HADD or the arm positioned in the sagittal plane, and (right) 90 of HADD or the arm positioned in the frontal plane.

All subjects performed manual isometric resistance exercises against external loads in 3 HADD angles in a quadruped position for EMG data collection: 1) 0° of HADD or the arm positioned in the sagittal plane, 2) 50° of HADD or the arm positioned in the scapular plane, and 3) 90° of HADD or the arm positioned in the coronal plane. The subjects were required to lift the exercise arm off the table during the quadruped position (Fig. 2). Isometric resistance exercises were implemented at 2 different shoulder abduction angles: 90° and 135° of ABD for 10 seconds each. The external load of 40% MMT were given just above the posterior portion of the elbow that was flexed in each of the 3 arm positions during the quadruped HABD resistance exercise, which was determined before the exercises for each subject. The amount of exercise intensity was selected as described by Bitter et al^3^^,^^6^ who demonstrated that the amount of infraspinatus EMG activity was significantly greater at 40% MMT with less contribution of MD and PD muscle activity than at 10% or 70% MMT in external rotation. The subjects were asked to match the manual resistance pressure given for each of the 3 HADD angles. Each subject performed all the manual isometric resistance exercises at 0° (or in the sagittal plane), 50° (or in the scapular plane), and 90° (or in the coronal plane) of HADD in each of the arm positions of ABD (90° and 135°) in a randomized order to minimize the systematic effect of motor learning and fatigue.

To clarify the exercise intensity of 40% MMT in the quadruped HABD isometric manual resistance exercise, this study included quadruped isotonic contractions with the same tested arm moving backward and forward about for 90° of HADD ranging from the sagittal to coronal plane and vice versa with the elbow joint flexed to 90°. The exercise was implemented with 2 different wrist cuff weights: 1.4 kg (3 lb) and 3.2 kg (7 lb), which was commonly used in shoulder rehabilitation exercises.^7^ Based on our pilot study, the cadence of the exercises was controlled by a metronome set at 75 beats per minute. The subjects performed rhythmical isotonic exercises with the weight load of 1.4 kg followed by the weight load of 3.2 kg for 10 times each. It was believed that this exercise protocol would help understand TMi EMG activity compared with IS and PD EMG activities.

### Data analysis

The EMG activities were collected using a data collection program (MP 150 Data Acquisition System; Biopac System, Inc., Goleta, CA, USA) with a sample rate of 1000 Hertz. All data were recorded and stored in a computer for off-line analysis. The mean EMG activity of the middle 2 seconds of each 4-second isometric contraction was calculated to determine the individual’s MVIC. For the isometric exercise condition, the mean EMG activity of the middle 5 seconds was calculated. All data were calculated in root mean square (RMS) values, normalized to MVIC of the corresponding muscles, and presented as a percentage of MVIC (% MVIC).

A 2 x 3 (ABD x HADD) repeated measures analysis of variance design within subjects crossed with arm positions was used to determine if there was any significant difference in mean values of normalized EMG muscle activity. A 1-way analysis of variance design was also used to determine any difference in the mean values of normalized EMG muscle activity across the rhythmical isotonic contractions with 2 different weight loads and isometric contraction at the coronal plane. Where appropriate, the simple main effect and a post hoc test with Tukey honestly significant difference were used to determine any significant difference. The level of significance was set at the 0.05 level.

## Results

The mean force values produced in the quadruped HABD at 90° and 135° ABD arm positions were determined as follows: 167.0 N and 157.8 N, respectively. Analysis of the results indicated no difference in the amount of the force the 90° and 135° ABD arm positions.

No significant interactions were observed for TMi, IS, TMa, and UT activities. However, significant main effects of HADD positions were observed for the marginal mean values of the 4 muscle activities (Table I). In addition, significant main effects of the ABD arm positions were observed for TMa activities (22.8 ± 15.4 and 16.9 ± 15.0% MVIC, 90° and 135° ABD, respectively) and for UT activities (15.0 ± 12.3 and 22.2 ± 12.1% MVIC, 90° and 135° ABD, respectively), while no significant main effects were observed between the 2 ABD arm positions for both TMi and IS activities.

**Table I:** The marginal mean values and standard deviations (in parentheses) of normalized EMG activity (% MVIC) of the teres minor, infraspinatus, teres major, and upper trapezius across in the 3 different arm position.

Muscle activity	Horizontal adduction			F ratio	*P* value	Critical
	0°	50°	90°			
Teres minor	15.5 (8.0)	26.2 (10.7)	41.4 (14.2)	146.4	<.001	3.71
Infraspinatus	8.6 (4.4)	13.0 (6.2)	19.2 (8.7)	68.1	<.001	2.62
Teres major	11.5 (7.9)	19.6 (13.0)	29.0 (18.2)	32.4	<.001	6.83
Upper trapezius	10.1 (9.1)	16.8 (9.1)	28.9 (11.8)	67.1	<.001	5.05

*% MVIC*, percentage of maximum voluntary isometric contraction; *EMG*, electromyography.

F ratios (2, 36) for each of the main factors and Tukey HSD critical values were shown for each of the differences in the mean values (*P* < .05).

Analysis of the results indicated a significant interaction between ABD and HADD angles for PD, MD, and LT activities (Table II). For SA muscle activity, no significant interaction or main factor of HADD positions was observed, while a significant difference was observed between 90° and 135° ABD (7.3 ± 5.1 and 10.9 ± 9.2% MVIC, 90° and 135° ABD, respectively).

**Table II:** The mean values and standard deviations (in parentheses) of normalized EMG activity (% MVIC) of the posterior deltoid, middle deltoid, and lower trapezius between 90° and 135° of abduction across at 0° of horizontal adduction (HADD) or the arm positioned in the sagittal plane, 50° of HADD or the arm positioned in the scapular plane, and 90° of HADD or the arm positioned in the coronal plane in the quadruped position.

Muscle activity	90° abduction			135° abduction			F ratio	Critical
	0°	50°	90°	0°	50°	90°		
Posterior deltoid	19.0 (5.0)††	30.1 (6.6)∗††	53.4 (12.5)††	24.3 (12.1)††	42.0 (17.2)∗††	55.4 (17.0)††	5.50	5.95
Middle deltoid	13.4 (3.9)∗††	25.4 (7.3)∗††	49.5 (14.5)††	22.7 (10.4)††	37.5 (14.3)∗††	52.6 (14.8)∗††	5.21	6.11
Lower trapezius	23.0 (7.7)∗†	28.9 (8.6)†	34.4 (14.3)∗††	14.1 (8.7)∗††	28.5 (10.7)††	44.2 (15.8)∗††	17.1	6.47

*% MVIC*, percentage of maximum voluntary isometric contraction; *EMG*, electromyography.

F ratios (2, 36) for each of the interventions for each of the muscle activities and Tukey HSD critical values were shown for each of the differences in the mean values for each of the muscles (*P* < .05). The asterisk (∗) indicates a significant simple main effect between 90° and 135° of ABD for each of the HADD angles (*P* < .05). The dagger (†) indicates a significant difference across the HADD angles for each of the arm position at 90° and 135° of ABD for each of the muscle activities with the critical value (*P* < .05).

The intensity of HADD manual resistance exercise with 40% MMT was identified as greater than that of rhythmical isotonic contraction exercise with the wrist cuff weight of 3.2 kg from the perspective of PD and MD muscle activities. As a result, the amounts of both TMi and IS muscle activities in the isotonic exercise were significantly less than those of manual resistance exercise (*P* < .05). Table III shows each of the mean values for muscle activities across isotonic exercises with the weight load of 1.4 kg and 3.2 kg and isometric contraction with the load of 40% MMT.

**Table III:** The mean values and standard deviations (in parentheses) of normalized EMG activity (% MVIC) of the eight muscle activities measured in the quadruped rhythmical concentric contraction with the wrist cuff weights of 1.4 and 3.2 kg and horizontal abduction manual resistance in the coronal plane with 40% manual muscle strength test (MMT).

Muscle activity	Isotonic contraction		40% MMT	F ratio	Critical
	1.4 kg	3.2 kg	Coronal plane		
Teres minor	25.1 (8.6)∗	32.8 (13.3)∗	41.2 (11.7)∗∗	12.7	8.3
Infraspinatus	12.4 (4.2)∗	14.1 (4.3)∗	18.5 (8.4)∗∗	12.8	3.2
Teres major	22.2 (12.7)∗	27.3 (12.8)	33.0 (16.0)∗	9.14	6.7
Posterior deltoid	31.5 (9.3)∗	37.4 (14.0)∗	53.4 (12.5)∗∗	25.4	8.3
Middle deltoid	27.5 (7.6)∗	33.2 (10.8)∗	49.5 (14.5)∗∗	20.3	9.2
Upper trapezius	27.2 (9.3)	33.0 (11.1)	26.3 (12.8)	3.28	
Lower trapezius	40.3 (13.1)	44.7 (13.4)∗	34.4 (14.3)∗	3.59	8.6
Seeratus anterior	8.8 (9.4)	8.6 (7.9)	6.3 (5.1)	0.35	

*% MVIC*, percentage of maximum voluntary isometric contraction; *EMG*, electromyography; *HSD*, honestly significant difference.

F ratios were shown for each of the main factors and Tukey HSD critical values were shown for each of the differences in the mean values (*P* < .05). The asterisk (*) indicates a significant difference in % MVIC across the quadruped rhythmical contractions with different weights and isometric contraction with 40% MMT in the coronal plane.

## Discussion

The findings of results were in line with the previous study in which TMi muscle activity was significantly increased during the standing 90/90 ER resistive band exercise when another resistive band was added to the distal portion of arm which augmented horizontal abduction resistance in the coronal plane, whereas it was not increased in the scapular plane.^27^ IS muscle activity is decreased in ER force as the arm is abducted whereas TMi muscle activity is not.^20^^,^^24^ Using positron emission tomography, Kurokawa et al^18^ identified that the TMi muscle generated higher muscle activity in ABD of the GHJ after ER exercise for 5 minutes in the supine position, while the IS muscle generated higher muscle activity in adduction of the GHJ. Moreover, Hamada et al^18^ compared the amount of TMi muscle activity between flexion and ABD of the GHJ without ER, and found that the maximum level of TMi muscle activity was progressively increased when the arm was flexed up to 120° or abducted up to 150°. They also confirmed greater activity of the TMi muscle in ER at 90° of GHJ ABD than in ER at 0° of ABD.

The findings of this study suggest that the IS muscle is not involved with HABD force as much as the TMi muscle at 90° of ABD. This might be attributed to different anatomical positions between the 2 muscles although the 2 muscles function as synergistic muscles of GHJ ER torque. The TMi muscle is innervated by the posterior axillary nerve, whose branch innervates the PD muscle, whereas the IS muscle is innervated by the suprascapular nerve.^2^^,^^4^^,^^19^ The TMi muscle inserts into the posteroinferior portion of the greater tuberosity aligned with the surgical neck of the humerus and from 3 to 5 o’clock, compared to the IS muscle, which inserts from 1 to 3 o’clock and is wrapped around the posterior aspect of the supraspinatus muscle insertion area.^9^^,^^15^ Thus, it is plausible to propose that the TMi muscle can be involved with the PD muscle to produce horizontal ABD, whereas the IS muscle is less likely involved with horizontal ABD at 90° of ABD.^27^

During surgical repair of the rotator cuff, only 1% of cases involved a repair of the TMi tendon, compared with the supraspinatus tendon which was involved in up to 75% of the total number of 581 rotator cuff surgeries in professional baseball players.^5^ Thirty-eight percent of the throwing athletes were only able to return to the same level of play or higher after rotator cuff repairs although 79% of the athletes were able to return to play.^1^ The occupational ratio of the TMi muscle can be increased as compensational hypertrophy in shoulders with tears in the supraspinatus and IS muscles.^17^ In addition, relatively high activity of the TMi muscle was observed during acceleration, deceleration, and follow-through phases of throwing, compared with the activity of IS muscle.^12^ Indeed, more than a half of female professional tennis players were able to play tennis with IS muscle atrophy whose mechanism was unknown on their dominant side.^33^ Furthermore, 4% of professional pitchers were identified to have IS muscle atrophy.^8^ Therefore, in addition to ER exercise in the shoulder adduction position commonly recommended for individuals with rotator cuff injuries,^23^^,^^32^ we recommend that individuals with symptomatic shoulders owing to habitual overhead performance include resistive exercise in 90° horizontal ABD at the coronal plane level.

Rhythmical isotonic contraction moving backward and forward between the sagittal and coronal plane with the wrist cuff weight of 3.2 kg did not activate the TMi muscle as much as manual isometric resistance at the coronal plane with the external load of 40% MMT. However, it significantly decreased MD muscle activity up to the moderate level,^12^ which might minimize the superior translation of humeral head.^3^^,^^6^ From the perspective of clinical implication, it would be beneficial for individuals with subacromial pain syndrome to perform quadruped isotonic movement or isometric resistance up to the scapular plane, which significantly decreased MD muscle activity as well.^29^

With regard to limitations, this study included a sample delimited to 1 cohort of collegiate baseball players, all of whom were highly trained to prepare for their upcoming season and had asymptomatic shoulders at the time of data collection. The subjects of this study may have developed TMi muscle strength as a result of their sports specificity and adaptation. Thus, the findings may limit the generalization regarding age and the level of performance to other populations and conditions.

This study used an active surface EMG electrode with a distance of interelectrode spacing of surface EMG of 10 mm which can reduce the contamination of crosstalk signals as compared with that of 22 mm.^10^ In addition, De Luca et al^11^ pointed out that the amount of EMG amplitude was much less in the musculotendon junction detected by the surface electrode than by the signal detected in the midline of the muscle belly. Thus, potential crosstalk between the TMi muscle and the long head of triceps brachii, located underneath the TMi muscle, appears trivial. In this study, both the TMi and TMa muscle were differently activated based on the results of this study, whereas we cannot completely remove the crosstalk signal from the PD muscle for the quality of TMi muscle activity measured using the surface EMG electrode.

## Conclusion

This study presented the careful selection of arm position that generates TMi muscle activity during the quadruped horizontal abduction exercise in the coronal plane more than that of the scapular and sagittal planes. Further studies are warranted to investigate TMi muscle activity to guide injury prevention and rehabilitation especially for overhead athletes.

## Disclaimers:

*Funding:* No funding was disclosed by the authors.

*Conflicts of interest:* The authors, their immediate families, and any research foundations with which they are affiliated have not received any financial payments or other benefits from any commercial entity related to the subject of this article.
